# Voices From the Community: Maternal Healthcare Experiences During the COVID-19 Pandemic

**DOI:** 10.7759/cureus.38323

**Published:** 2023-04-30

**Authors:** Tejbeer Singh, Ravneet Kaur, Shashi Kant, Kapil Yadav, Sanjeev Gupta

**Affiliations:** 1 Centre for Community Medicine, All India Institute of Medical Sciences, New Delhi, New Delhi, IND

**Keywords:** experiences during covid-19, health service utilisation, maternal healthcare services, facilitators and barriers, covid 19, qualitative research

## Abstract

Introduction

The coronavirus disease 2019 (COVID-19) pandemic had a significant impact on health services around the world. Many hospitals and clinics were overwhelmed by the influx of patients, leading to delays and disruptions in care. The fear of contracting the virus also led to a decrease in the number of people seeking medical care, even for urgent or life-threatening conditions. Various studies have reported a decrease in overall utilization of maternal health services. However, it remains vital to find the reasons for reduced utilization along with the experiences of the women as well as healthcare workers during the pandemic.

Objective

The objective of this study was to identify the facilitators and barriers to maternal healthcare services utilization during the COVID-19 pandemic.

Methods

It was a qualitative study conducted in a rural area of Haryana, India. Twelve in-depth interviews (IDIs) were conducted with health workers and four focused group discussions (FGDs) were conducted with pregnant women. Textual analysis was done for both IDIs as well as FGDs. Qualitative analysis was done manually.

Results

The identified themes were complete cessation of services, no outpatient department (OPD) services for many months, no antenatal care (ANC) services for two months, disruption of supply of medicines, unavailability of drugs, fear of getting COVID-19 infection, mandatory COVID-19 negative report for admission in hospital, and increased referral from government health facilities during the pandemic and lockdown.

Conclusion

Maternal healthcare services suffered during COVID-19 for various reasons including the closure of health facilities, limited supply of stocks, or fear of the disease among pregnant women. This evidence can be used to prepare as well as manage healthcare services in future.

## Introduction

Coronavirus disease 2019 (COVID-19) was declared a pandemic on March 11, 2020 [1], and in India, a lockdown was imposed from March 24, 2020 [2]. The COVID-19 pandemic was largely characterized by widespread lockdowns to prevent infection spread [3].

The COVID-19 pandemic had a significant impact on health services around the world. Hospitals and clinics were overwhelmed by the overflow of patients, leading to delays and disruptions in care. In some cases, non-emergency medical procedures were postponed or cancelled to prioritize more urgent cases. This caused concern among pregnant women, who were worried about their ability to access the care they needed during this difficult time [4,5]. As a result, it was more important than ever for pregnant women to utilize these services to ensure the health and safety of both themselves and their unborn children.

Furthermore, the pandemic also led to overburdened and limited staff in healthcare facilities, as some healthcare workers fell ill or were quarantined. This further strained the ability of these facilities to provide timely and effective care to all patients as well as pregnant women [6,7]. Additionally, the fear of contracting the virus also led to a decrease in the number of people seeking medical care, even for urgent or life-threatening conditions. This resulted in a decrease in the overall utilization of maternal health services, which could have serious consequences for pregnant women who may be at higher risk for complications [8]. Overall, the COVID-19 pandemic had a significant impact on health services, leading to delays, disruptions, and reduced access to care for many patients, including pregnant women [9].

The COVID-19 pandemic had a significant impact on antenatal services, as many pregnant women were hesitant to seek care due to concerns about contracting the virus. A study conducted in Delhi, India, reported that there was a significant decline in the utilization of antenatal visits during the pandemic, with a decrease of up to 80% [10]. It also reported that pregnant women who did seek care experienced longer wait times and delays in receiving the care they needed. This could have serious consequences, as timely and regular antenatal care is crucial for the health and well-being of both the mother and the fetus/newborn.

Various studies were conducted to find the proportion of maternal healthcare services utilization or reduction in utilization [11-13] but not much is known about the real-life experiences of the women or health workers in rural India. So, it remains crucial to find out the reasons which lead to change in the utilization of maternal healthcare services, its facilitators and barriers, and the experiences of women during that period.

This study was conducted with the objective of identifying the facilitators and barriers to maternal healthcare services utilization during the COVID-19 pandemic.

## Materials and methods

This was a qualitative study conducted in the Ballabgarh block of the Faridabad district in Haryana, India. The study area comprised 28 villages covered under the intensive field practice rea (IFPA) of the Comprehensive Rural Health Services Project (CRHSP), Ballabgarh. It consisted of the two primary health centres (PHCs), namely, PHC Chhainsa and Dayalpur. Each PHC included six Sub-Centres covering a total population of around 1,00,000. The detailed site description is provided elsewhere [14]. The study duration was two months, from December 16, 2020, to February 7, 2021. The study was conducted after obtaining the approval from Institute Ethics Committee for Post Graduate Research, All India Institute of Medical Sciences (AIIMS), New Delhi, India (approval number: IECPG-634/25.11.2020). Written informed consent was taken from all the participants for interviews as well as audio recordings, and a Participant Information Sheet (PIS) was provided. All collected information was kept confidential.

Study population and sample size

There was a total of 39 participants included in this study.

Pregnant women registered in the Health Management Information System (HMIS) were chosen from four villages purposively, so that the participants had either their antenatal, natal, or postnatal period during the COVID-19 lockdown (March 25, 2020, to May 31, 2020) and were deemed likely to have experienced the effects of lockdown on healthcare services while trying to avail them. There were 27 such participants.

There is usually one female healthcare worker from AIIMS, New Delhi, at each Sub-Centre, and they were part of the study as a participant. If in case there were two health workers at any Sub-Centre who were working for AIIMS, New Delhi, then we chose only one out of them. So, from each Sub-Centre under the two PHCs, Dayalpur and Chhainsa, one health worker was selected, that is, a total of 12.

Sampling strategy

We conducted an in-depth interview (IDI) with each health worker from each Sub-Centre. Thus, 12 IDIs were conducted among the 12 health workers.

A total of four focussed group discussions (FGDs) were conducted among the participants in four villages namely, Chhainsa, Dayalpur, Machgarh, and Jawan. In Dayalpur, it was conducted in an Anganwadi centre, whereas in Jawan and Machgarh, FGDs were conducted in PHC Sub-Centres. In Chhainsa, it was conducted at the PHC campus. The audio of the interview was then transcribed and domains were identified, which were further categorized into themes on the basis of the results obtained.

The study instrument was a topic outline guide for IDIs and FGDs. Topic outline guide for IDIs included: the perception of health workers regarding the status of maternal healthcare services during the COVID-19 lockdown, supply and distribution of drugs during COVID-19 lockdown, problems faced by pregnant women in availing maternal healthcare services during the lockdown, and measures adopted to provide services during the lockdown. The topic outline guide for the FGDs included questions regarding: the availability of maternal healthcare services during the lockdown, problems faced in availing maternal healthcare services during the lockdown, and perceived barriers and facilitators in accessing maternal healthcare services during the COVID-19 pandemic.

Analysis

Textual analysis was done for both IDIs and FGDs. Data for IDIs and FGDs were analyzed manually. Themes were identified after free listing and domain evaluation. Validity was achieved by triangulating the findings of FGDs and IDGs from Auxiliary Nurse Midwives (ANMs), who worked as health workers in the Sub-Centres. The analysis was done manually.

## Results

Socio-demographic profile of the participants

A total of 12 IDIs were conducted among healthcare workers. The details of the key informants (health workers) are shown in Table 1.

**Table 1:** Key informant details MPW: Multipurpose Worker; HA: Health Assistant: RCH: Reproductive and Child Health

S. No.	Place	Position/Job	Experience in RCH services (in years)
1	Chandawali	MPW	17
2	Nawada	MPW	20
3	Machgarh	MPW	17
4	Shahpur Kalan	HA	21
5	Garkhera	MPW	17
6	Dayalpur	MPW	14
7	Atali	HA	21
8	Ladholi	MPW	17
9	Naryala	MPW	21
10	Jawan	HA	23
11	Fatehpur Billoch	MPW	19
12	Chhainsa	HA	21

Four FGDs were conducted in four villages, Dayalpur, Machgarh, Chhainsa, and Jawan among the 27 women participants. The number of participants from Dayalpur and Chhainsa was eight each; there were six from Machgarh and five from Jawan. The mean age of participants was 24 years, 40% of participants were educated up to the secondary level, and 33% of participants were educated up to the primary level. Out of the total, 85% of participants belonged to above poverty line criteria and 37% belonged to Scheduled Castes and Scheduled Tribes (Table 2).

**Table 2:** Socio-demographic profile of the participants *Economic status was assessed based on the availability of Below Poverty Line (BPL) cards issued by the Government of India. **SC: Scheduled Caste, ST: Scheduled Tribe

Variable		Frequency (percentage) (N=27)
Age group (years)	<25	13 (48.2)
	25-29	12 (44.4)
	>30	2 (7.4)
Education	Graduation & above	5 (18.5)
	Secondary	11 (40.7)
	Primary	9 (33.3)
	Illiterate	2 (7.4)
Economic status*	Above Poverty Line	23 (85.2)
	Below Poverty Line	4 (14.8)
Caste	SC/STs	10 (37.0)
	Others	17 (63.0)
Family	Nuclear	17 (63.0)
	Extended	10 (37.0)

Facilitators and barriers to maternal healthcare services during the pandemic

Status of Maternal Healthcare Services during the COVID-19 Pandemic

There was a complete cessation of services from April 1, 2020, to May 31, 2020. To prevent the spread of infection, health services provided in Sub-Centres and PHCs were stopped. The key informants told that during lockdown, almost all services were stopped, be it house visits, immunization, or investigations during health facility visits. After one and a half months, the immunization services resumed. Participants also said that they found the Sub-Centres closed for around one month during the lockdown. Later, they started receiving the services. This finding was corroborated by the following verbatims:

“No services were provided for a month, neither investigation was done nor Tetanus Toxoid (TT) was given. After that we started working” (Health worker from Chandawali)

“My check-ups were not done for one month; then they started doing it" (Participant 4, Chhainsa)

“Then after 1.5 months we got orders to start vaccination. So, we again started calling everyone through Accredited Social Health Activists (ASHAs)” (Health worker from Chandawali)

“Here (at the Sub-Centre), we were refused, so we used to go to private (healthcare facility).” (Participant 2, Jawan)

The health workers revealed that they could not identify new high-risk pregnancies and they then only tried to focus on already registered high-risk pregnancies. They could not conduct house visits due to the lockdown as ANC and outpatient department (OPD) services were stopped for many months and ANC services resumed after around four months.

“High risk was not detected. We focused on those who we already knew.” (Health worker from Naryala)

“OPD was started in December. ANC and vaccination was already started; vaccination was started after one month while ANC started after four months.” (Health worker from Garkhera)

Supply and Distribution of Drugs During the COVID-19 Pandemic

The health workers highlighted that the supply of drugs was limited during the lockdown and many essential drugs were not available during that period, even iron-folic acid (IFA) tablets were not available, which is one of the most important supplements for every pregnant woman. In the FGD, participants too said that they did not receive any tablets during the lockdown. In a few Sub-Centres, where IFA stock was available, IFA distribution was not affected due to COVID-19. So, they did manage regardless of the supply of medicines.

“There was no supply, but we already had stock, so there was no difference due to the lack of supply. We had IFA tablets. Calcium tablets were not already being provided, so it was not there.” (Health worker from Naryala)

“I did not get any tablets, for some time they denied that the centre was closed, later tablets were not available there.” (Participant 5, Chhainsa)

Barriers to Accessing Maternal Healthcare Services

One major barrier was the fear of getting COVID-19. As there was havoc due to the reported severity of infection or the unknown effects of the virus on the fetus, everybody was afraid of getting infected by COVID-19. This fear led to missing ANC visits among pregnant women. The healthcare facilities were keen on the availability of the COVID-19 test report before admitting any patients. This was a barrier to accessing health services during that time. This finding is supported by the quotes of health workers.

“There was fear that we might get COVID-19. Even the hospitals were not admitting patients without COVID-19 (test) report. First thing was to ask for COVID-19 report.” (Health worker from Nawada)

During that period, most government hospitals had a prerequisite of a negative COVID-19 report; so if someone was not having it, they were either referred or had to wait until the report was available. This led to delays in accessing appropriate services, which had some serious consequences, i.e., there was one maternal death due to a delay in managing postnatal complications. For this reason, many women avoided visiting government health facilities and instead preferred private healthcare facilities for childbirth. One health worker insisted on the same fact while stating that there was no increase in home deliveries.

“There was also a maternal death. First, she was taken to Tigaon in their own vehicle (could not arrange an ambulance). Then after keeping her for some time, she was referred from there. But because she was not having a COVID-19 test report with her, nobody admitted her” (Health worker from Nawada)

“Her (a patient) condition was very serious and she was referred to B.K. (Badshah Khan Hospital), from B.K. to Safdarjung Hospital. No one was willing to examine her because of COVID-19 (non-availability of a negative COVID-19 test report).” (Participant 3, Machgarh)

“There was no home delivery here, but more deliveries happened in private.” (Health worker from Atali)

Efforts to Provide Services During Lockdown

During the lockdown, to provide services to pregnant women, ASHA workers used to contact them on their mobiles to ask for information and even visited their homes while following all precautionary methods. IFA was provided during the visits by ASHA if the pregnant women were unable to visit the healthcare facility.

“The ASHA worker used to visit some homes or used to ask by phone only." (Health worker from Machgarh)

"The visits were few, and few people used to come here. Pregnant women used to take medicines from the house of the ASHA worker.” (Health worker from Machgarh)

**Figure 1:**
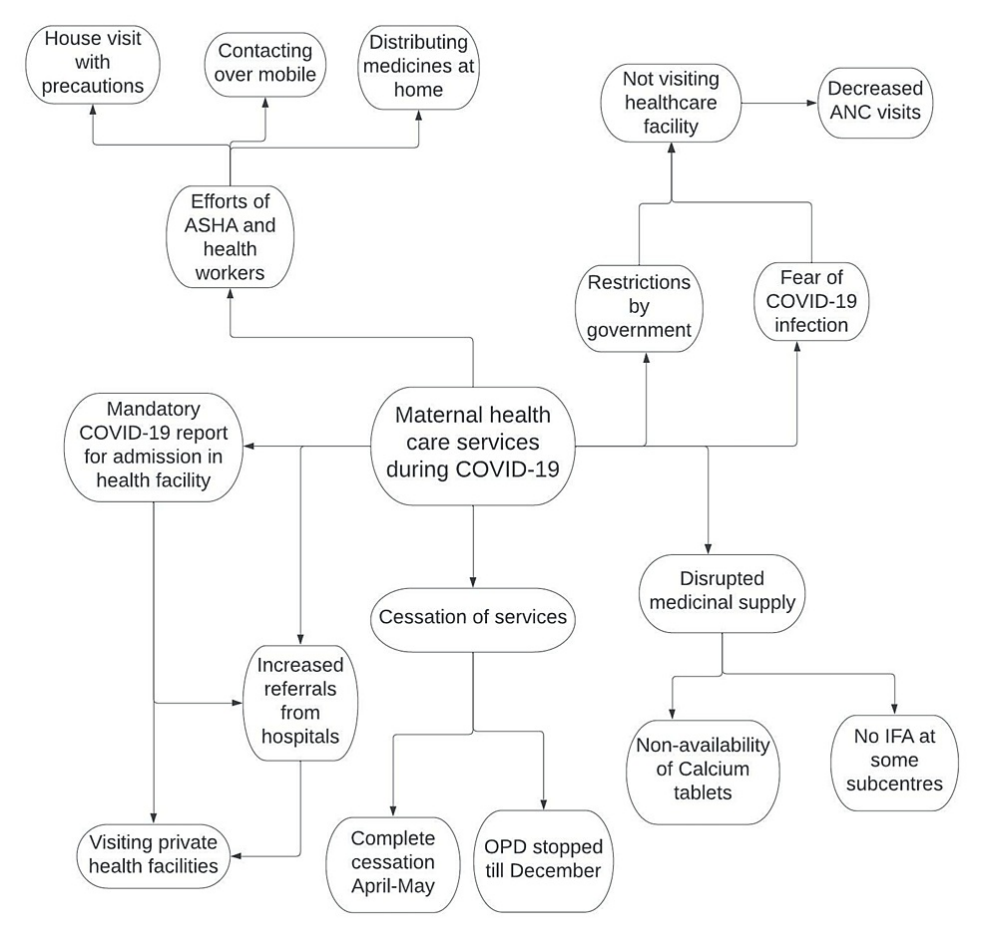
Facilitators and barriers to maternal healthcare services during COVID-19 OPD: Outpatient Department, COVID-19: Coronavirus Disease 2019, ANC: Antenatal care, ASHA: Accredited Social Health Activist, IFA: Iron and Folic Acid

The findings of the IDIs and FGDs are summarized in Table 3 and Table 4, respectively. The facilitators and barriers to maternal healthcare services are shown in Figure 1.

**Table 3:** Domains and themes identified in IDIs OPD: Outpatient Department, COVID-19: Coronavirus Disease 2019, ANC: Antenatal care, PNC: Postnatal care, ASHA: Accredited Social Health Activist, IDI: In-depth Interview

S. No.	Domain	Theme	Description
1	Status of Maternal Health Care services	Complete cessation of services for one and a half months	No services were being provided for nearly one and a half month because of lockdown, No ANC/PNC visits No investigations (couldn’t give appropriate number of IFA tablets because did not have Hb report) No new registrations No immunization services No high-risk pregnancy identification
		OPD services affected for many months while ANC services affected for nearly two months	ANC clinic and Immunization was resumed in June 2020 While OPD services started in December 2020
2	Supply and distribution of drugs during COVID-19 lockdown	Disruption of supply of medicines	Supply of drugs was limited with complete absence at some subcentres, while some subcentres got supply and some already had stock of medicines. Calcium tablets were already not in supply even before COVID-19 but supply of IFA tablets was disrupted during lockdown
3	Barriers to access Maternal Health Care services	Fear of getting COVID-19 infection	They could come to subcentre but not to other places for which they had to travel outside the village, because they were scared that would get COVID-19 Hospitals too asked for COVID-19 report before admitting any patient
		Mandatory COVID-19 Negative Report for admission in hospital	Women were being referred for delivery from government hospitals if they were not having COVID-19 report
4.	Facilitators for providing services during lockdown	Persistent efforts by ASHAs for continuation of services	At some subcentres ASHAs used to go for HBPNC visits, followed all precautionary measures. ASHAs used to contact patients over phone, provided update to the ANM about new pregnancies. Distributed tablets at their homes when needed.

**Table 4:** Domains and themes identified in FGDs COVID-19: Coronavirus Disease 2019, ANC: Antenatal care, FGD: Focussed Group Discussion

S. No.	Domain	Theme	Description
1	Status of maternal healthcare services during COVID-19 lockdown	No services for one month	For around one month no services were being provided. Some women had to find another facility for ANC visits.
		Unavailability of drugs	Tablets were not being provided. Because either the facility was closed or there was a lack of supply
2	Problems faced in availing maternal healthcare services	Increased referral from Government health facilities and going to private hospitals	Women were referred because of COVID as nobody was preferring to examine. Some women preferred to go to private hospital for delivery because of COVID-19
3	Barriers for availing services	Fear of COVID-19	The women did not go for checkups because they were afraid of getting COVID-19 infection themselves They also feared that it may harm the baby as well

## Discussion

This study highlights the challenges and facilitators for providing maternal healthcare services during the COVID-19 pandemic.

It was found that there was a complete cessation of all services for one and a half months during the first wave of COVID-19 in April and May 2020. During that time no ANC, postnatal, OPD, or immunization services were provided, which can explain the reduced utilization of maternal services during that period [15]. A study done in the Netherlands also reported a reduction in ANC services utilization during the period [16].

Many factors were responsible for the reduced utilization of maternal healthcare services during the pandemic. Strict restrictions on movement, unavailability of transport, closure of health facilities, to name a few. Studies done in other parts of the world also found similar barriers while considering maternal healthcare services utilization as, throughout the world, lockdowns were imposed to prevent the spread of COVID-19 [5,17-19]. There was fear of getting infected with COVID-19 among pregnant women so they avoided visiting healthcare facilities for their ANC visits. This was confirmed by other studies [20-22].

The supply of medicine/supplements was also limited during that time, and while some Sub-Centres did not receive any medicines or supplements at all, others got a limited supply. Some Sub-Centres had a stock of maternal healthcare-related medicines (IFA, calcium tablets) and so they managed. IFA and calcium are supplements recommended for every pregnant woman and their unavailability would put health programs back to square one. These findings are similar to other studies [6,23,24]. A study conducted in Pakistan also supported our finding [25]. Since the prevalence of anaemia has been increasing among pregnant women over the years [26], any shortage in providing IFA would only add to the burden.

Women who visited health facilities for childbirth were asked about their COVID-19 test report, which was made mandatory. If someone didn’t have it, then they were asked to get it before admission, so most of the participants, instead, visited private healthcare facilities. This led to an increased proportion of deliveries in private institutions during that period. A similar finding was reported in another study [27].

This study found that frontline workers such as ASHAs played a major role in the provision of services during the pandemic. In the IFPA, ASHAs provided services in the community by calling pregnant women and following up over the phone, distributing IFA tablets to those who needed them, and doing house visits following COVID-19-appropriate behaviour. This was an important facilitator in providing some important services, which was sustainable throughout the pandemic. Bankar and Ghosh reported similar findings [28]. 

The strengths of the study are the community-based qualitative study design, which captures the real-life experiences of the participants during the pandemic and the rural setting as very few studies were done to identify the issues faced in rural India during the pandemic. This study had a few limitations: we did not collect information regarding teleconsultations. Interviews with medical officers at PHCs could have been conducted to better understand the sequence of events during lockdown while closing facilities or resuming services.

## Conclusions

During the COVID-19 pandemic, maternal healthcare services suffered along with other healthcare services. The unavailability or low supply of drugs/supplements required during pregnancy such as IFA and calcium may have adverse effects on maternal health such as anaemia. High-risk pregnant women could not be identified during the pandemic, which meant that were they to develop complications later, it would be unexpected and put a strain on the existing health system. The mandate of COVID-19 reports before admission adds to the challenges faced by pregnant women. These findings can be used during the planning and management of health policies in future. Healthcare facilities need to be prepared for such challenges. Facilitators for service provision such as efforts made by healthcare workers and ASHAs should be appreciated. 
